# Gelation Behavior and Stability of Multicomponent Sterol-Based Oleogels

**DOI:** 10.3390/gels8010037

**Published:** 2022-01-05

**Authors:** Artur J. Martins, Fátima Cerqueira, António A. Vicente, Rosiane L. Cunha, Lorenzo M. Pastrana, Miguel A. Cerqueira

**Affiliations:** 1 International Iberian Nanotechnology Laboratory, Av. Mestre José Veiga s/n, 4715-330 Braga, Portugal; fcerqueira@fisica.uminho.pt (F.C.); lorenzo.pastrana@inl.int (L.M.P.); miguel.cerqueira@inl.int (M.A.C.); 2 Centre of Biological Engineering, Campus de Gualtar, University of Minho, 4710-057 Braga, Portugal; avicente@deb.uminho.pt; 3 Centre of Physics, Campus de Gualtar, University of Minho, 4710-057 Braga, Portugal; 4 Department of Food Engineering, Faculty of Food Engineering, University of Campinas, UNICAMP, CEP, Campinas 13083-862, Brazil; rosiane@unicamp.br

**Keywords:** confocal Raman spectroscopy, oil structuring, self-assembly, γ-oryzanol, β-sitosterol, oleogelator

## Abstract

Novel fat mimetic materials, such as oleogels, are advancing the personalization of healthier food products and can be developed from low molecular weight compounds such as γ-oryzanol and β-sitosterol. Following molecular assembly, the formation of a tubular system ensues, which seems to be influenced by elements such as the oleogelators’ concentration and ratio, cooling rates, and storage periods. Sterol-based oleogels were formulated under distinct environmental conditions, and a comprehensive study aimed to assess the effects of the mentioned factors on oleogel formation and stability, through visual observation and by using techniques such as small-angle X-ray scattering, X-ray diffraction, confocal Raman spectroscopy, rheology, and polarized microscopy. The long, rod-like conformations, identified by small-angle X-ray scattering, showed that different cooling rates influence oleogels’ texture. Raman spectra showed that the stabilization time is associated with the interfibrillar aggregation, which occurred differently for 8 and 10 wt%, with a proven relationship between ferulic acid and the tubular formation. This report gives fundamental insight into the critical point of gelation, referring to the time scale of the molecular stabilization. Our results verify that understanding the structuring mechanisms of oleogelation is decisive for the processing and manufacturing of novel foods which integrate oleogels in their structure.

## 1. Introduction

In past years, attempts have been made to replace saturated and hydrogenated (trans) fats from processed food products [1,2,3,4,5]. The push for the introduction of oleogels in foods has seen significant developments, foreseeing the benefits of their solid-like properties, good mouthfeel attributes, and bioactivity capabilities [6]. Under certain conditions, supramolecular low molecular self-assemblies evidence enough oil structuring capabilities, producing oleogels through solvent entrapment processes which develop robust three-dimensional networks [7,8,9,10]. Active research on molecular self-assembly mechanisms has been made in past years. Such occurrences can arise spontaneously or be influenced by external factors (e.g., shear, ionic charges, temperature, light) [11,12,13,14]. The application of low molecular weight gelators (LMOGs) has been gaining attention, due to the gelation mechanisms and also because of the existence of new food-grade ingredients with gelling capability [15]. Bot and Agterof reported on γ-oryzanol and phytosterol combinations, showing that sterols (e.g., β-sitosterol, cholesterol, dihydrochlesterol, ergosterol, stigmasterol, cholestanol) can associate with γ-oryzanol to form oleogels [16,17]. These plant sterol-based oleogels are recognized for their attractive optical and textural properties. Molecular dynamic modeling simulations corroborate that γ-oryzanol and sterol esters self-assemble into hollow tubular conformations, developing interconnected non-covalent bonds (van der Waals and π−π connections) between the fibrils. Studies on gel development and combinations of β-sitosterol (or other sterols) with γ-oryzanol appraised the influence of the oleogelator ratio on gel morphology [14,16], concluding that the 1:1 molar ratio of oryzanol: sitosterol produced oleogels with the highest firmness values [18]. Light has been shed on the effect and behavior of these mixtures throughout the structuring period, at different length scales and from a chemical point of view with the support of small-angle X-ray scattering (SAXS), atomic force microscopy (AFM), X-ray diffraction (XRD), and Fourier-transform infrared spectroscopy (FTIR) [17,18,19,20,21,22,23]. However, until now, Raman spectroscopy has not been used to evaluate how temperature and oleogelator ratios truly impact the oleogelation mechanism. Comprehensive reviews of Raman spectroscopy on lipids, oil types, and other naturally occurring fats (vegetable and animal sources) have been published [24,25]. However, these were mainly focused on component analyses through sample correlation with fatty acids and structural assignments. Raman scattering applied to polymer-based (ethylcellulose) oleogels reported on the stretching vibrations of the CH2 band as a result of polymer conformation sensitivity [26]. Sterol-based systems exhibit an erratic gelling performance, without unprecise gelling periods under quiescent conditions, where a small mechanical commotion can trigger gelation [17]. Deprived of such disorder, is safe to say that the gelling time is not predictable and intermittent gelling behavior can constrain its applicability. To face this problem, we aimed to better describe the gelling performance, using different oleogelator concentrations, ratios, and colling rates. The impact of the interfibrillar aggregation was studied over time by confocal Raman spectroscopy and complemented with SAXS, XRD, polarized microscopy, and texture analysis. It is our expectation that this study will supplement the current knowledge on the gelation mechanisms of the phytosterols–oryzanol system, adding valuable information towards understanding changes within the different structural levels, ranging from the nano-, micro-, to macroscale.

## 2. Results and Discussion

### 2.1. Small-Angle X-ray Scattering

**Figure 1:**
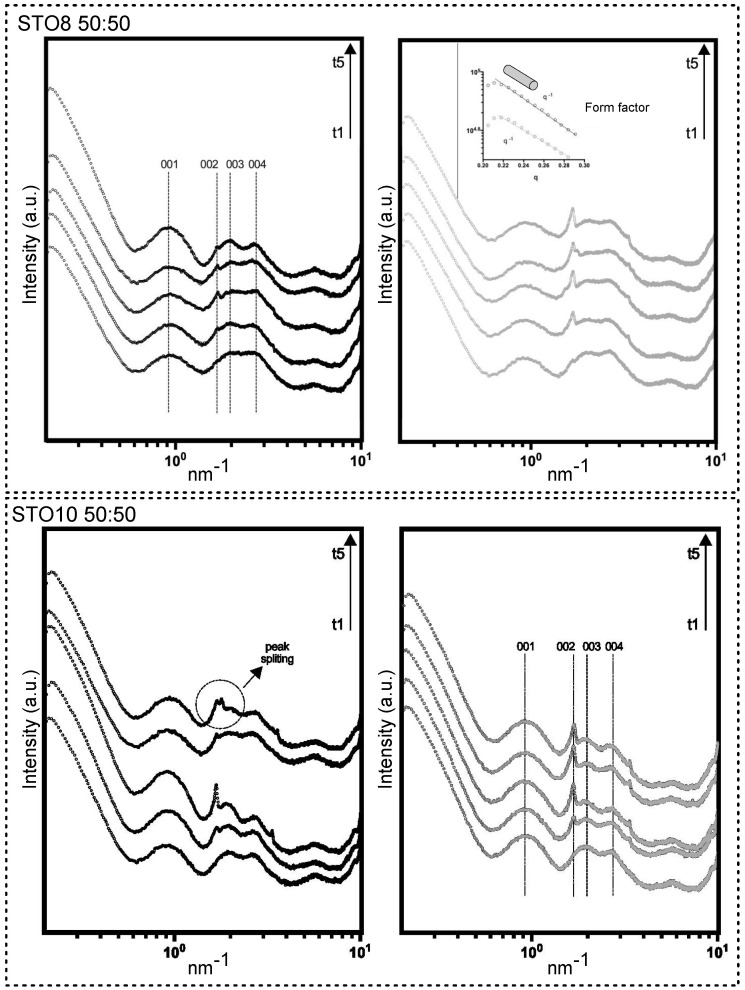
SAXS patterns for 50:50 samples produced under ramp r1 and r2 (**left** and **right**, respectively). Time points are displayed in growing order (bottom to top) from t1 to t5, where t1 = 24 h; t2 = 7 days; t3 = 2 weeks; t4 = 3 weeks; t5 = 28 days (4 weeks). Reflections displayed with (001), (002), (003), and (004).

A broadening view of small-angle X-ray scattering (SAXS) provided information on the size, shape, and stability of molecular arrangements. The model-dependent analysis in the lower-angle region defined the form factor of a rod-like cylinder (molecular shaping characterized by −1 slope in the lower q, for all samples). *d* (nm) = 2 * pi/*q*, where *q* identifies the wave vector peak position, related to precise diffraction. Sharp-like features were spotted (whole spectra); however, pronounced progress of the structural arrangements was observed through storage for the 50:50 samples (Figure 1). Diffractions after *q* = 1 nm^−1^ fared to sharpen for longer storage times. The 60:40 samples unveiled noteworthy stability associated with the inexistence of structural modifications. Unaltered Bragg peaks were present for the 60:40 oleogels (Figure S1). Spacings for the 50:50 oleogels at *d* = 6.72 nm (001), *d* = 3.26 nm (002), *d* = 3.17 nm (003), and *d* = 2.32 nm (004) were detected. A higher structural definition was induced through faster cooling (r2). The distance between the center points of the cylinders remained constant, regardless of the cooling ramp and oleogelator concentrations [27]. Matheson et al. [20] reported on density escalation (number) and extension of the (nanometric) tubules which resulted from the increase in the structuring agent mass. This ensued with no effects on the cross-section of the self-assemblies, not influencing transparency and hardness, as will be discussed further. A broad initial peak is characteristic of tubules shaped in an oil medium, starting from approximately 40% of the oil fraction [28]. Despite the subsequent increase under r2, both samples (through time) still exhibited a peak position ratio that is characteristic of hexagonal symmetry, with lattice packing relative to the position of the first peak resembling the 1, √3, 2, √7 orders [27,28]. Peak position ratio stability was observed for all samples, confirming that the self-assembled tubules persisted for all compositions. No additional diffractions outside of the hexagonal geometry (e.g., lamellar or cubic packing) were attributed. This could, however, occur with variations in concentration (e.g., if the oil content was lowered) [29]. The decrease in oil content and the increase in gelator concentration could affect lattice diffraction; despite the packing interference, more resolved peaks were observed, yielding a long-range ordering [30]. A peak splitting event in the hexagonal packing, between *d* = 3.26 and 3.17 nm at t5 for STO10 50:50-r1 (Figure 1), can explain the interfiber aggregation phenomena, resulting in the opacity for the 50:50 gels richer in sitosterol content [18].

It was observed that, during storage, the formed macroscopic sterol crystals tend to grow bigger (Figure S2). No major variations in the aromatic ring and ferulic acid moiety modes were perceived as a consequence of gelator ratio variation. The ferulic acid was hypothesized as being responsible for keeping the nanotubes apart, inducing the tilted stacking of the molecules [7], as seemed to be corroborated in our work.

### 2.2. X-ray Diffraction (XRD)

Small diffraction peaks were visible on certain XRD patterns, ranging between 14.5 and 15.5° approx. (Figure S3). This lattice relates to *d*-spacings between 5.77 and 6.11 Å, close to the reported crystalline conformations of β-sitosterol in oil [31]. No consequences on the crystalline packing, resulting from the different cooling ramps, were detected. Broad diffraction peaks (2*θ*) were found around 18/20° with corresponding spacings ranging from 4.49 to 4.74 Å. All the samples presented similar patterns, except for some intensity variations. Comparable diffractions to the ones previously observed for oleogels produced with pure oryzanol and sitosterol were identified [8,31]. The nonexistence of a long-range translational crystalline ordering is perceptible through the absence of any sharp features, as observed previously for oleogels produced with the same oleogelators [28]. The preservation of this signaling during storage is indicative of the increased stability of this system over time.

### 2.3. Confocal Raman Spectroscopy

**Figure 2:**
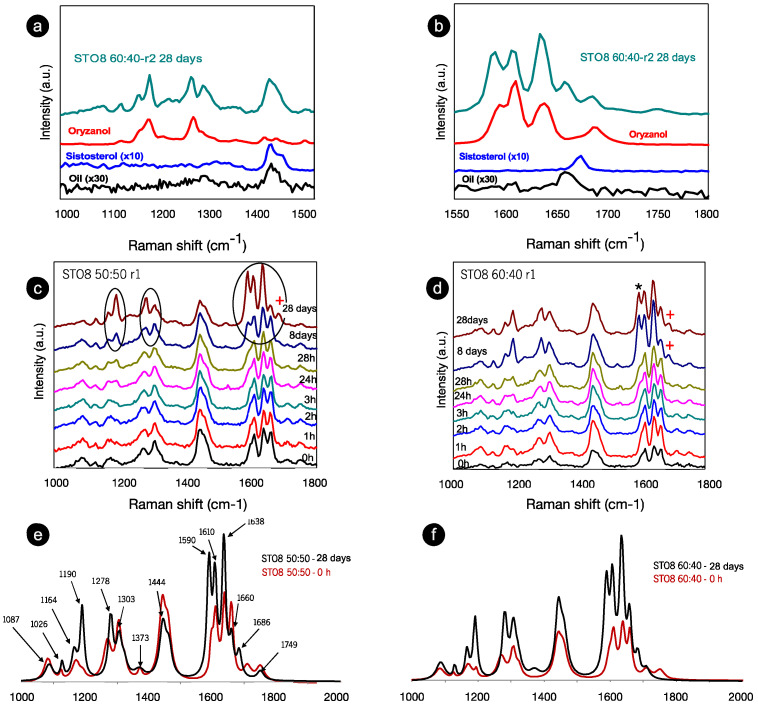
Room temperature Raman spectra for (**a**) a range of 1000–1550 cm^−1^ and (**b**) a range of 1550–1800 cm^−1^ of the recorded spectra for the oleogelator powders (γ-oryzanol and β-sitosterol), oil sample (HOSO), and oleogel sample (STO8 60:40) after 28 days. (**c**) STO8 50:50-r1 and (**d**), STO8 60:40-r1 at different storage times. (**e**,**f**) represent the corresponding first (0 h) and last (28 days) recorded spectra; mode at ≈1590 and ≈1686 cm^−1^ marked with (*) and (+) respectively.

The confluence between oils, structurants, and gelation mechanisms has been targeted by emerging approaches, exploiting complementary techniques such as molecular dynamics simulations and spectroscopy methods [19,26,27]. To further investigate the molecular nano-incidences on gelation performance, confocal Raman spectroscopy was used. After production, Raman spectra were acquired at different storage times (from 0 h to 28 days); STO8 oleogels spectra (Figure 2) recorded the response for two oryzanol–sitosterol ratios under r1 cooling rate for the 1000–1800 cm^−1^ range (range I: 1000–1400 cm^−1^ and range II: 1500–1800 cm^−1^).

In this range are vibrational modes associated with C-C, C-O, C-H, C-H2, C=C, and C=O bonds (either stretching, twisting, wagging, or deformation vibrations). These relate to lipid species due to a highly non-polar association with highly polarizable C-H and C-C bonds [32]. After 28 days, the components’ contributions during the ST08 60:40 oleogel development stage (Figure 2a,b) were observed, and despite the shift of the small peak position, the main contribution is derived from the γ-oryzanol, whereas the sitosterol’s influence was only seen in the 1440 cm^−1^ modes. Moreover, the intensity ratio between oryzanol-related features in the component and the oleogel sample (at the same storage time in the 1550–1800 cm^−1^ range) is dissimilar—clear evidence of the γ-oryzanol contribution, indicative of the level of influence attributed to the other components in the end conformation. The spectra for the two proportions of oryzanol–sitosterol are similar until the eighth day (Figure 2c,d). Despite the comparable spectra at 0 h and 28 days for the two ratios (Figure 2e,f), the time progression at 8–28 (Figure 2c,d) did not follow the same pattern. During this period the spectra were identical for the 60:40 samples, resulting in a faster stabilization. Major changes were identified for the 8 wt% 50:50 sample between the 8th and 28th days—significant (circled) changes in range I (Figure 2c). These changes can be summarized as follows: (i) the transformation of wide bands into (well defined) peaks; (ii) a change in intensity ratio between modes (in some cases there is even an inversion of intensity ratios); (iii) a slight shift in some peak positions. These changes were at least observed at the endpoint, independently from sterol concentration, gelator ratio, and cooling rate. For the 1000–1500 cm^−1^ range, the most significant changes mentioned and highlighted are the increased definition (clear after 28 h) and intensity of the ~1190 cm^−1^ mode (with respect to the 1164 cm^−1^ mode), a slight peak shift of the mode at 1278 cm^−1^, and its intensity inversion (with respect to the 1303 cm^−1^ mode). However, the most relevant changes occurred within the 1500–1800 cm^−1^ range, recapped by an intensity increase at 1635 cm^−1^ and two well-resolved modes at ≈1590 and ≈1686 cm^−1^, marked with (*) and (+), respectively, in Figure 2d. The 1686 cm^−1^ mode is new, whereas the 1590 cm^−1^ existed as a shoulder at 0 h for both ratios. The spectra (from Figure 2a,b) were fitted and the positions of the peaks, full width at half maximum intensity, were obtained, as summarized in Table 1.

**Figure 3:**
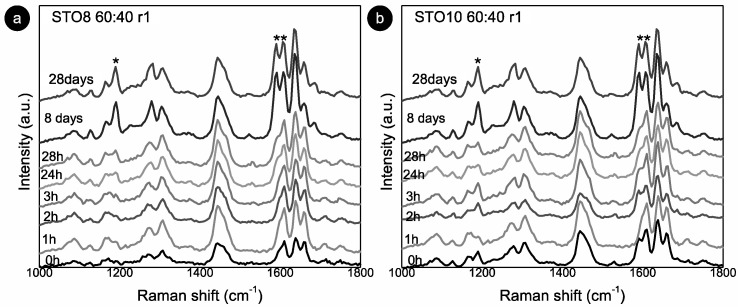
Room temperature Raman spectra for: (**a**) STO8 60:40-r1 and (**b**) STO10 60:40-r1. (*) and (**) represent modes at 1190 cm^−1^ and 1590 cm^−1^ respectively.

The 1190 and 1164 cm^−1^ modes relate to the oryzanol ferulic acid moiety (associated with the methoxy group) [33] and also to the aromatic C-H in-plane bending/deformation (possibly shifting from 1176 to 1189 cm^−1^) [34]. The peak at 1164 cm^−1^ relates to C-OH stretching (attached to the aromatic ring of the ferulic acid), while the roughly unchanged mode at 1444 cm^−1^, is linked with β-sitosterol and HOSO (pointing to CH2 scissoring vibrations). Despite small peak position shifts, the 1278 cm^−1^ and 1590 cm^−1^ modes are clearly also γ-oryzanol-bound, the first being attributed to the aromatic C-H deformation and the latter to the stretching vibrations, C=C, of the aromatic ring. The ”new” 1686 cm^−1^ mode is prevalent in the γ-oryzanol spectrum. Dalkas et al. reported similar changes for >5 wt% gels (equal ratio), associating variations with the interfibrillar aggregation [21]. Those observations are linked to the strong intensity increase at ~1190 cm^−1^ as a result of oleogelator concentration, mentioning a prominent band at ~1672 cm^−1^ as characteristic of cholesterol and a group of β-sitosterol-like sterols (which retain the four interconnected cycloalkane groups, sitosterol being one of them) [25]. This band originates from the C=C stretching vibrations of the aromatic ring and is not independently visible for the oleogels. This is explained by the arrangement of the two molecules in an oil medium, generating a “weakening” effect, alongside the ones detected for γ-oryzanol and oil in the same region. For the STO10, the mode related to the aromatic ring stretching vibrations C=C (marked with **) is detected at 0 h (whereas for the 8 wt% it is perceived at 24 h), revealing a faster configuration of the tubular assemblies. Hence, for this concentration, the stacking of the oryzanol and sitosterol molecules occurs at a faster pace and, consequently, the stability of the tubules is reached quicker. Despite some intensity variations (Figure 3b), the mode at 1190 cm^−1^ (*) is quite explicit right from the beginning, while for STO8 it only becomes relevant after 8 days, settling the faster stabilization for the first. Concerning the Raman signature, there is no distinction between the ramps used.

**Figure 4:**
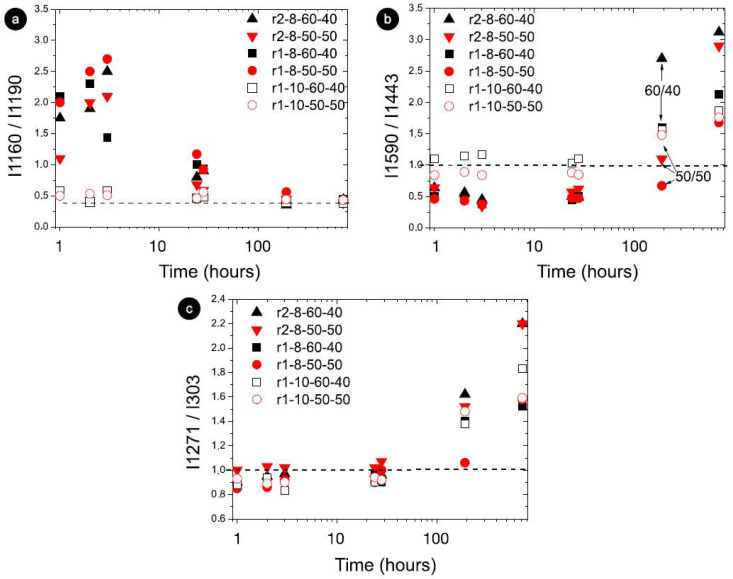
Raman modes with the most intensity variation as a function of storage for STO8 samples, and for r1-STO10 (50:50 and 60:40); 10 wt% (open symbols) and 8 wt% (full symbols); red and black colors represent 50:50 and 60:40 ratios, respectively. xx axis on a logarithmic scale. The horizontal dashed line indicates (**a**) the final condition reached; (**b**,**c**), the value 1 for the intensity ratio (meaning equal intensity).

Raman spectra evidenced the same structure, modes, and generally the same behavior. However, time-supported alterations were detected between the two cooling rates, being further apparent for the 50:50 ratio (Figure S4). An intensity inversion during storage on Raman modes, mainly between 24 h and 8 days (192 h) of storage, was noted. Hence, greater transformations ensued in this period as follows: (i) faster gelation of the 10 wt% oleogels with equal initial and final states, whereas the 8 wt% oleogels reach the final state only after 8 days (Figure 4a); (ii) the faster gelation of the samples with a 60:40 ratio (Figure 4b); (iii) the faster gelation, without oleogel deterioration, under the r2 cooling rate (Figure 4c).

The intensity ratio analysis showed significant changes, namely at the inversion of modes 1160 cm^−1^ and 1190 cm^−1^ (I1160/I1190) in Figure 4a. For the 10 wt% samples, the initial intensity ratio was essentially the final one, steering towards faster gelation, as illustrated in Figure 4 for fundamental storage times, and different intensity ratios between 1 and 3 for 8 wt% and around 0.5 for 10 wt%. The storage effect on the intensity ratio of modes 1590 cm^−1^ and 1440 cm^−1^ displayed an inversion of the intensity (Figure 4b) from a < 1 value (initially) to a > 1 value (at the end). The ratio effect on gelation time (Figure 4b) suggested that the primary gelling stage was reached earlier for the 60:40 samples (mass effect illustrated in the initial time points). The time performance seen for I1271/I1303 exposed the intensity inversion under r2, demonstrating a steeper and faster inversion compared with r1 (Figure 4c). As recent reports showed, the bundling effect on oleogel structure would be influential on the hardening and stabilization of the oleogels [35,36]. As mentioned earlier, the interfibrillar aggregation is decisive, and the way that the bundle formation (size and number of bundles) occurs and evolves over time is part of the multi-step oleogelation process. Reports showed changes in bundle arrangement where fibrils aggregate differently depending on structuring agent concentrations as well as in the presence of polar components [20,35]. This could be largely influenced not only by the oil medium and gelator concentration and ratio but also by cooling rates.

Features within the 2600–3200 cm^−1^ range are emphasized; however, minor spectra variations were observed (Figure S5). The characteristic modes of the symmetric and antisymmetric (C-H) vibrations were observed for (most abundant in edible oils) methyl (CH3) and methylene (CH2) terminal chains [24]. The weak intensity at ~3010 cm^−1^ (HOSO carbon chain UFAs) was revealed to be constant for all samples [24]. Reports on oil oxidation allude to the feature at 2875 cm^−1^ (unaffected by oxidation) as a spectral normalization to distinguish the oxidative features. This was not explored here, since the spectra are not well defined and nearly no changes arose in this range. According to Machado et al., the superposed bands between 2900 and 2950 cm^−1^ should escalate upon oxidation [37]. This behavior was constant for all samples (Figure S5a) when compared to virgin HOSO. Remarkably, in this range, the Raman spectra for HOSO were essentially the same at 0 h and at 30 days. However, the unstructured spectrum, with bands relating to superposed peaks, did not allow any further conclusions. Previous arguments on the oxidation of edible oils (e.g., linolenic acid) have been reported [38], discussing the intensity variations of the 1635 and 1695 cm^−1^ modes (non-existent here)—the 1695 cm^−1^ mode is assigned to C=O, referring to conjugated aldehydes [37]. Additionally, the mode around 1596 cm^−1^ is recognized as a result of oxidation products [37]. Despite the absence of the mode at 1695 cm^−1^, two additional ones were visible within the vicinity of the 1635 and 1596 cm^−1^ modes, (~1639 cm^−1^ and 1590 cm^−1^). Nonetheless, these were just seen in the γ-oryzanol spectrum (Table 1 and Figure 2b), and despite the noteworthy progressive evolution, such cannot be associated with oxidative processes. Heated in the same fashion as the oleogels, virgin HOSO and HOSO samples (28 days-Figure S5b) did not reveal the 1635, 1695, and 1596 cm^−1^ modes, attributed to oxidation [37]. The Raman spectroscopy of oils can be a source of information on unsaturation levels [25,26,38,39,40]. Relevant peaks report to (i) C=O vibrations (1750 cm^−1^) with high intensity attributed to SFA—for oleogel samples, this mode was either absent or exhibited low intensity; (ii) C=C olefinic molecule vibrations (1660 cm^−1^), for which the intensity decrease relates to a higher unsaturation degree—for oleogels, the intensity of this mode decreases with time; (iii) C-H vibrations (1440 cm^−1^) are seen as a pattern for UFAs—we observed no intensity variations; (iv) C-H and C-H2 vibrations (1260 and 1300 cm^−1^) at 1260 cm^−1^ refer to CH deformations of cis-double bonds and would decrease its intensity upon oxidation, originating from cis-double bond obliteration (intensity ratio linked to unsaturation level)—for oleogels, the intensity of this mode decreases with time and 1260 cm^−1^, compared to the t1300 cm^−1^ mode, increases in intensity in the same period.

### 2.4. Polarized Microscopy

**Figure 5:**
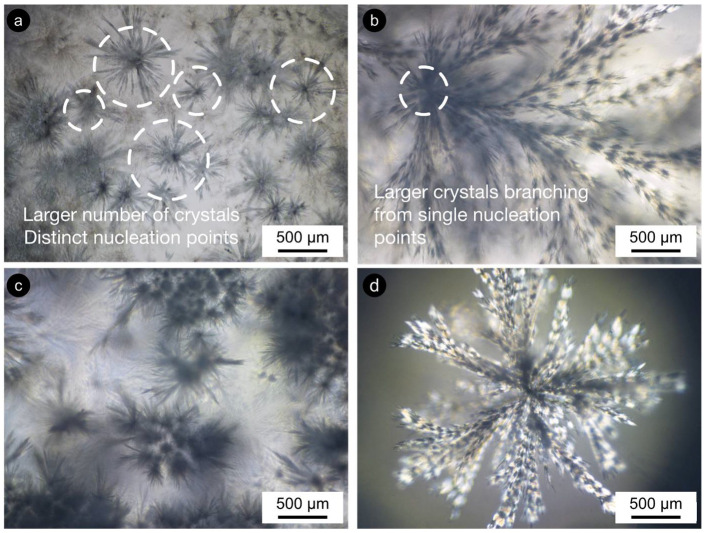
Crystal growth morphology/disposition for STO8 50:50 under (**a**) r1 = 1 °C·min^−1^ and (**b**) r2 = 7 °C·min^−1^ (both after 24 h); (**c**) r1 = 1 °C·min^−1^ and (**d**) r2 = 7 °C·min^−1^ (both after the first week). Micrographs obtained under polarization with 50× magnification.

Co-crystallization developed micro- and macroscopic crystalline bodies (Figure S2). These seem to relate to the excess of β-sitosterol, which was unable to bridge with γ-oryzanol at the nanoscale. The changes in the 50:50 samples weren’t so clear for STO10 as they were for STO8 (Figure 5 and Figure S2). Crystallization for STO8 varied with time and the ramified crystalline structures (dendrites) also differed, morphology-wise. Less pronounced changes were visible for STO10 50:50-r1 as the ones developed under r2. The crystal sizes increased; however, a lower upsurge in crystal numbers was spotted for r1 samples when compared to r2. A similar tendency was detected for the r2-treated sample; however, higher cooling rates seemed responsible for a distinctive effect on the deceptive larger number of nucleation points. The effects on crystal morphology, conveyed by the cooling rates, were fairly visible for STO8 50:50 (Figure 5c and Figure S2). STO8 50:50-r2 produced larger ramified fibrils evolving from fewer nucleation points until reaching the container wall or other crystal branches. STO8 50:50-r1 unveiled smaller crystals, coined from distinctive points, and some managed to aggregate into clusters (Figure 5d).

**Figure 6:**
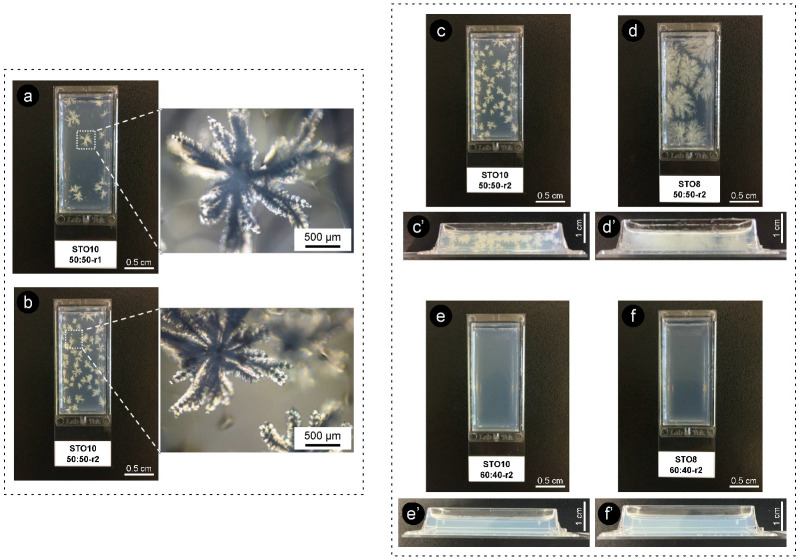
Images of oleogels obtained under different cooling ramps after 2 weeks of storage (r1 = 1 °C·min^−1^ and r2 = 7 °C·min^−1^). On the left are also images obtained by polarized microscopy. (**a**) STO10 50:50-r1, (**b**) STO10 50:50-r2, (**c**) STO10 50:50-r2, (**d**) STO8 50:50-r2, (**e**) STO10 60:40-r2, (**f**) STO8 60:40-r2. (**c’**–**f’**) show the respective cross-section view.

The quite evident influence of the cooling rate for equal ratio samples has shown that, for 8 wt% oleogels under r2, multiple conformations ensued and exhibited fine dendrites (radiating from a smaller population of nuclei), while for r1, an increased number of nucleation sites formed minor dendrite bodies (Figure 5 and Figure 6). All oleogels were fairly transparent at 4 h, though it was still verified that higher sterol concentrations disclosed some (not measured) opacity/haziness, which can be credited to the increased density of the nanostructured tubules (Figure S2). As a result of the cooling rate, small crystalline conformations were identified by polarized microscopy (at the same storage time) for the STO8 50:50-r1 sample, which was not observed for STO8 50:50-r2. It was not possible to observe a similar response in STO10. After 8 h, variations in STO 8 50:50-r1 did not occur for other samples. Oleogels produced with a 60:40 ratio presented high morphological stability over time, with no crystalline bodies observed. In contrast, the STO10 50:50 samples (under both cooling ramps) exhibited larger crystal sizes and density, where a single crystal morphology from several nucleation points was detected (Figure 6). From dissimilar circumstances, plentiful crystalline conformations evolved, being such hylotropic crystal occurrences similar to the ones reported with γ-oryzanol and cholesterol [39]. For the 50:50 ratio, there is an excess of sitosterol, which led to the observed macroscopic differences in STO8, impacting differently on STO10 samples (Figure 6). The lack of hylotropic balance reduced the system miscibility for the 50:50 samples, contributing to a decrease in nucleation sites (in comparison to the 60:40 samples), resulting in the creation of larger crystalline bodies.

Distinctive energies of activation result from ratio variations, influencing nucleation and consequent crystal growth [14]. The major recognized trend between the STO8 and STO10 (50:50 ratio) samples relied on crystal growth disparities, even under similar cooling rates. Higher oleogelator concentrations produced dense and less ramified structures, as opposed to the thinner, highly ramified crystallographic bodies observed for lower concentrations (Figure 6d). The 60:40 samples remained translucid, exhibiting a slight haziness after 7 days; this feature was sustained during the following days. Limited system miscibility was accentuated as an outcome of the increasing concentration effect, justifying the visual differences between STO8 and STO10 produced under the same cooling ramp, r1. This generated lesser nucleation points while increasing inaccurate fibrilar orientation.

### 2.5. Texture

Compression until break tests confirmed that cooling rates led to distinctive hardness values just after 28 days of storage (Figure S6). An opposing effect at the endpoint revealed a lower hardness for oleogels produced under r2. Such textural behavior is justified by the unveiled nanometric stability of the tubular conformations, which was achieved faster for STO10 60:40, agreeing with the results from the Raman analysis, as the final spectra conformation was reached faster. STO8 and STO10 (60:40) displayed solid conformations, at least at the 3 h time point (for both cooling ramps), while the 50:50 gels exhibited similar visual performance at the 24 h time point. The Raman analysis pointed towards differences in the critical point of gelation (referring to the molecular stabilization) between 28 h and 8 days, for STO8 60:40-r1, STO10 60:40-r1, and STO10 50:50-r2. The critical point of gelation for STO8 50:50-r1 occurred between the eighth day and the endpoint. The non-covalent interfibrillar connections, composed of van der Waals and π−π connections among the ferulic acid groups of γ-oryzanol, led to the connection of the fibrilar building blocks in the architectural arrangement, meaning that storage time and sterol concentrations influenced the molecular vibrational arrangement, and, hence, the texture.

### 2.6. Rheology-Induced Structuring

**Figure 7:**
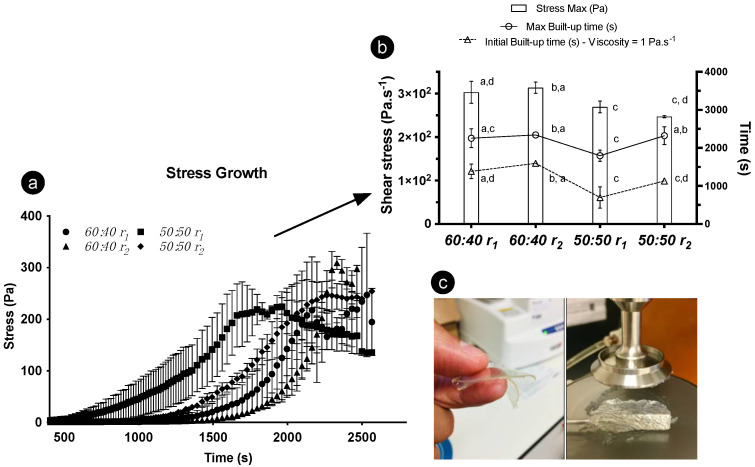
Rheological results for 8 wt% oleogels; γ-oryzanol-β-sitosterol with 50:50 and 60:40 ratios were tested. (**a**) Stress growth curves; (**b**) stress max, max build-up, and initial build-up data gathered after using different cooling ramps (r1 = 1 °C·min^−1^ and r2 = 7 °C·min^−1^); (**c**) gels obtained through rheology.

The divergent structural build-up derived over time and its impact on the gelation period was measured under two cooling ramps (using two ratios and one concentration of oleogelators). With increasing time, higher stress values were registered until reaching the maximum stress point (Figure 7a). Such a response occurred as the gel viscosity increased due to the formation of a 3D fibrilar architecture, not exclusively triggered by the temperature decrease but also interconnected with time, as a contributor to the hardening of the gel. The structuring procedure can be prompted through shear movement, though it is unclear if molecular arrangements, at least for the 60:40 formulations, would develop equally at the micro- and nano-scales as they would under steady conditions. Significant differences in max and initial build-up time were witnessed, as the 50:50 sample subjected to r1 displayed a quicker build-up under the rheometer probe (Figure 7b). This can be interconnected to different molecular arrangements and separate crystallization, resulting in gelling network variations [18]. We found that these differences induced faster-paced gelation for some samples, consequently influencing their rheological properties. Likewise, the cooling ramps to which the oleogels were exposed before the stress test influenced the definitive rheological response.

## 3. Conclusions

With the support of techniques such as SAXS [17,18,20], AFM [20], XRD [19], FTIR [41], modeling simulations [21], and rheology as a measure for texture development (hence gelation), advances in the study of the supermolecular assembly of sterol based-oleogels have been achieved. Despite the recently intensified discussion [42], additional insights on such erratic gelation conditions have proven to be necessary, and we have provided these by detailing the observed time-dependent molecular engagements. Our findings showed that the micro and nanometric stability of the tubular self-assembled system is intertwined with aspects such as oleogelator ratios and concentrations, as well as the cooling rates and storage period. X-ray studies demonstrated the increased stability of this system over time with the constancy of the rod-like cylinder form factor despite cooling ramps and oleogelator concentrations. Disparities in the critical point of gelation were detected by a Raman analysis, referring to the molecular stabilization of the samples between 28 h and 8 days for STO8 60:40-r1, STO10 60:40-r1, and STO10 50:50-r2. This feature appeared to occur between 8 days and the final time point for STO8 50:50-r1. Different conditions allowed for the development of distinct crystalline conformations, where an excess of sitosterol led to differences with regard to nucleation site development as well as for the ramifications observed for STO8. The STO10 60:40 oleogels exhibited solid behavior at the 3 h time point under both cooling ramps, and equal visual properties were only identified for the STO 50:50 oleogel at 24 h. Despite this, cooling ramps and oleogelator concentrations impacted the hardness of the oleogels, influencing the non-covalent interfibrillar connections (ferulic acid groups of γ-oryzanol and both the hydroxyl and methoxy groups). The higher firmness in the 60:40 ratio oleogels (comparable to 1:1 molar ratio) corroborated earlier results [18,43]. We identified a contrasting behavior between the first and the final time point, since a faster cooling ramp revealed a hardness profile prone to reaching higher values, symptomatic of a delayed stabilization effect that could lead to increased hardness after some time. Bot et al. exposed the importance of the induced shear on the gelation of sterol-based oleogels, reporting an immediate structuring event after cooling [16,17]. We can now sustain that the molecular stabilization of these gels was conceivably unfinished, hence a more resolved gel structuring would differently impact the oleogels’ end properties, with greater influence on the ones produced with lower oleogelator concentrations. Identical reasoning should apply for combinations of γ-oryzanol with other phytosterols and different oil mediums (different solubilities) which could reveal dissimilar self-assembly mechanisms. The oryzanol ferulic acid group was reported to be decisive in the stability-driven interfibrillar bundling networks [21]; although, time-dependence was not evaluated before. Confocal Raman spectroscopy endorsed an improved understanding of the evolution of the metastable liquid state under quiescent conditions, allowing for the connection of the self-assembly mechanism to the physical and chemical properties of the oleogels, advancing the knowledge in colloid and interface science, deepening the understanding of the gelation in a sterol-based oleogel system, and substantiating the relevance of the nano-scale occurrences in micro- and macro-scale properties. This study reveals the need for a multi-analytical approach along the development stage to better understand the impact of the above-mentioned aspects in the technological properties of the oleogels.

## 4. Materials and Methods

### 4.1. Oleogel Production

High oleic sunflower oil (HOSO) composed by 0.1% of C14:0, 3.8% of C16:0, 3.3% of C18:0, 80.1% of C18:1, 10.7% of C18:2, 0.3% of C18:3, 0.4% of C20:0, and 0.1% of C20:1, was offered by Cargill (Brazil). γ-oryzanol was acquired from Oryza Oil and Fat Chemical Co. Ltd. (Ichinomiya, Japan) and β-sitosterol > 70% from Sigma (St. Louis, MS, USA). Samples were independently produced by heating the mixture of HOSO and oleogelators at 85 ± 5 °C for 30 min under stirring. The ratios and oleogelator concentrations were selected upon consideration of the literature, seeing a 1:1 molar of oryzanol–sitosterol as the formulation responsible for generating the firmest oleogels [18]. Since β-sitosterol > 70% was used, a 60:40 ratio of oryzanol–sitosterol was explored first to resemble the adequate molar ratio. Later, concentrations of 8 and 10 wt% of an oryzanol:sitosterol mixture (STO8 and STO10) were used for the development of oleogels with ratios of 50:50 and 60:40 (STO8 and STO10). After production, the samples were poured into glass slide containers (40 mm × 20 mm × 10 mm). The curing process was done in a Weiss Technik WKL 34/70 Climatic chamber (Grand Rapids, MI, USA) under two cooling rates (r1 = 1 °C·min^−1^ and r2 = 7 °C·min^−1^). Samples were stored at room temperature (22 ± 2 °C) prior to further analysis.

### 4.2. Small-Angle X-ray Scattering

SAXS measurements were performed using an Anton Paar SAXSess mc2 model (Anton Paar, Graz, Austria), operating at 40 kV and 50 mA at ~22 °C. Representative sections of the oleogel samples were placed in TCS sample stages used for solid measurements (Kapton scattering recorded no interference). Data were collected with an image plate detector (2D data acquisition).

### 4.3. X-ray Diffraction (XRD)

An X-ray Diffractometer X Pert PRO MRD from Malvern Panalytical Ltd. (Royston, UK) was used for X-ray diffraction analysis (XRD) during the different time points. X-ray scans were done at room temperature (~22 °C) in the range of 10 to 50° (2*θ*°), using a Cu source, the X-ray tube (γ = 1.54056 Å) at 45 kV and 40 mA with *θ* set to −0.0372 degrees for fine calibration offset. Diffraction parameters were obtained from the minimum of the 2nd derivative with parameters set for peak search in HighScore Plus software. The lattice parameter d was determined using Bragg’s law, Equation (1), where λ is the wavelength of the X-ray used, *θ* is the half of the diffraction Bragg angle (2*θ*) and d is the space between planes.

n·λ = 2d·sin *θ* (1)

### 4.4. Confocal Raman Spectroscopy

An Alpha300R confocal microscope, (WITec) with a 532 nm Nd:YAG laser was used for Raman spectroscopy measurements. A *p* = 2.5 mW laser beam was focused on the samples with a ×50 lens (Zeiss) with a numerical aperture (NA) of 0.9 at a Z value of approx. 2200 µm. The spectra were collected in the 80–3500 cm^−1^ range, with 600 groove/mm grating using 10 acquisitions with a 2s acquisition time (150 acquisitions for oil samples). After baseline correction, spectra were fitted with Lorentzian functions. From the fitting, the peak position and FWHM were obtained for each mode. Vertical shifts were performed to improve data comprehension. The relevant Raman information falls in the ranges of 1000–1800 cm^−1^ and 2700–3100 cm^−1^.

### 4.5. Gelation Observation and Rheological Study

Oleogels with 8 wt% (above critical gelation concentration) were chosen due to increased susceptibility to external parameters and were produced using 50:50 and 60:40 ratios (oryzanol:sitosterol). Morphological evolution was monitored and rheology was carried out with STO8 gels. Oscillatory temperature (cooling) ramps of 1 and 7 °C.min^−1^ were executed after erasing the oleogel structural history. Stress growth tests were performed due to oleogels’ rheopectic nature when stationary (before hardening), monitoring the structural build-up (at room temperature) with a constant shear-rate in a Discovery Hybrid Rheometer (DHR1) from TA Instruments (New Castle, DE, USA), equipped with a 40 mm parallel plate. A two-step procedure was carried out, comprising an oscillation-temperature ramp from 80 °C with a resting time of 10 s and ending at 22 °C with a sampling interval of 10 pt s^−1^, strain fixed at 0.1 %, and a 0.1Hz frequency. The stress growth profile was obtained at room temperature (22 ± 2 °C) at 3000 s with a shear rate of 10 s^−1^.

### 4.6. Polarized Microscopy

Oleogels were analyzed at room temperature with a polarized light microscope (Olympus System Microscope model BX51TF, Olympus America Inc., Center Valley, PA, USA) equipped with a digital camera (Olympus EX300, Olympus America Inc., Center Valley, PA, USA). Pictures were taken at a magnification of 50× and 200×.

### 4.7. Texture

Compression textural experiments were performed in a Texture Analyzer TA-XT2i (Stable Microsystems, Surrey, UK) equipped with a 30 kg load cell with a P25 SMPS probe. Five replicates of each formulation with an 8 mm diameter and 8 mm height were performed. Test conditions involved a compression speed of 1.0 mm·s^−1^ (with the same speed of probe pulling) with a maximum strain of 50%. Such a strain value is able to perform fractural damage, as desired, obtaining an overall response of the gel hardness.

### 4.8. Statistical Analysis and Data Visualization

Analysis of variance, through Tukey’s mean comparison test (*p* < 0.05), was performed in Prism 9 (GraphPad Software, Inc., San Diego, CA, USA). Rheology device control and the calculation of rheological parameters were performed under TRIOS Software, Version 4.1.1.33073 (TA Instruments, New Castle, DE, USA). The SAXSquant and SaxsQuant2D software package (Anton Paar GmbH, Graz, Austria) were used to control the SAXS device, the data acquisition, and the normalization of the SAXS profile masks after profile integration. X’Pert HighScore Plus software (PANanalytical, Almelo, The Netherlands) was used to gather XRD data, collected at 174 s, and to perform peak diffractions analysis. Hardness was calculated using the Texture Exponent v.6.1.1.0 software (Stable Microsystems, Godalming, UK).

## Figures and Tables

**Table 1:** Registered peaks for the Raman Spectra of STO8 60:40-r2 at 28 days, γ-oryzanol and β-sitosterol powders, and the HOSO sample at 0 h and at 28 days.

STO8 60:40-r2 28 Days (cm^−1^)	γ-Oryzanol (cm^−1^)	β-Sitosterol (cm^−1^)	HOSO0 h (cm^−1^)	HOSO 28 Days (cm^−1^)
1749	-	-	1742	1740
1706	-	-	-	-
1686	1689	1674	-	-
1660	-	-	1655	1655
1639	1639	-	-	-
1610	1610	-	-	-
1590	1593	-	-	-
1509	1520	-	-	-
1456	1459	1463	1463	1460
1444	1434	1446	1446	1442
1373	1375	-	-	-
-	1324	1335	-	-
1305	1302	-	1305	1305
1278	1283	-	-	1264
-	-	-	1270	-
1227	1220	-	-	-
1190	1188	-	-	-
1164	1168	-	-	-
1126	1130	-	-	-
1087	-	-	-	1087
-	-	-	1080	
-	-	-	-	1065
-	1044	-	-	-

(-) no peak was found in the vicinity of the selected compound.

## Data Availability

The data presented in this study are available on request from the corresponding author.

## Supplementary Materials

The following are available online at https://www.mdpi.com/article/10.3390/gels8010037/s1. Figure S1: SAXS patterns collected to all STO samples; Figure S2: Pictures and polarized micrographs of oleogel samples; Figure S3: XRD spectra of STO samples; Figure S4: Raman spectra for STO8 samples produced with both cooling ramps; Figure S5: Raman spectra for oleogel and oil samples; Figure S6: Hardness values of oleogels.
