# Supplementary material for: Gelation Behavior and Stability of Multicomponent Sterol-Based Oleogels
Source: Gels. 2022 Jan 5;8(1):37. doi: 10.3390/gels8010037 (PMC8774356; doi:10.3390/gels8010037)
Supplement: Supplementary file 1 [file gels-08-00037-s001.zip › gels-1529726-supplementary.pdf]

gels

MDPI

Supplementary Material

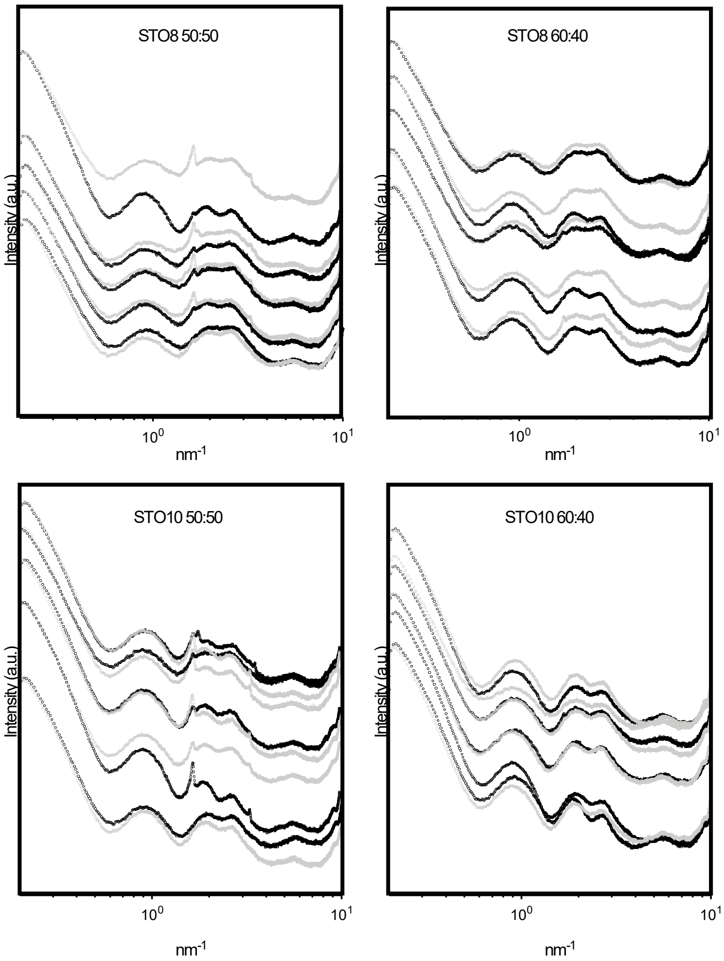

Figure S1. SAXS patterns collected to all STO samples during the storage period. Different points are displayed in growing order (bottom to top) from t1 to t5, where t1 = 24 h; t2 = 7 days; t3 = 2 weeks; t4 = 3 weeks; t5 = 28 days (4 weeks); r1 and r2 data are displayed in black and grey, respectively. Data was collected during 28 days and the curves have been shifted vertically for easier observation and were placed in ascendant order (t1 to t5).

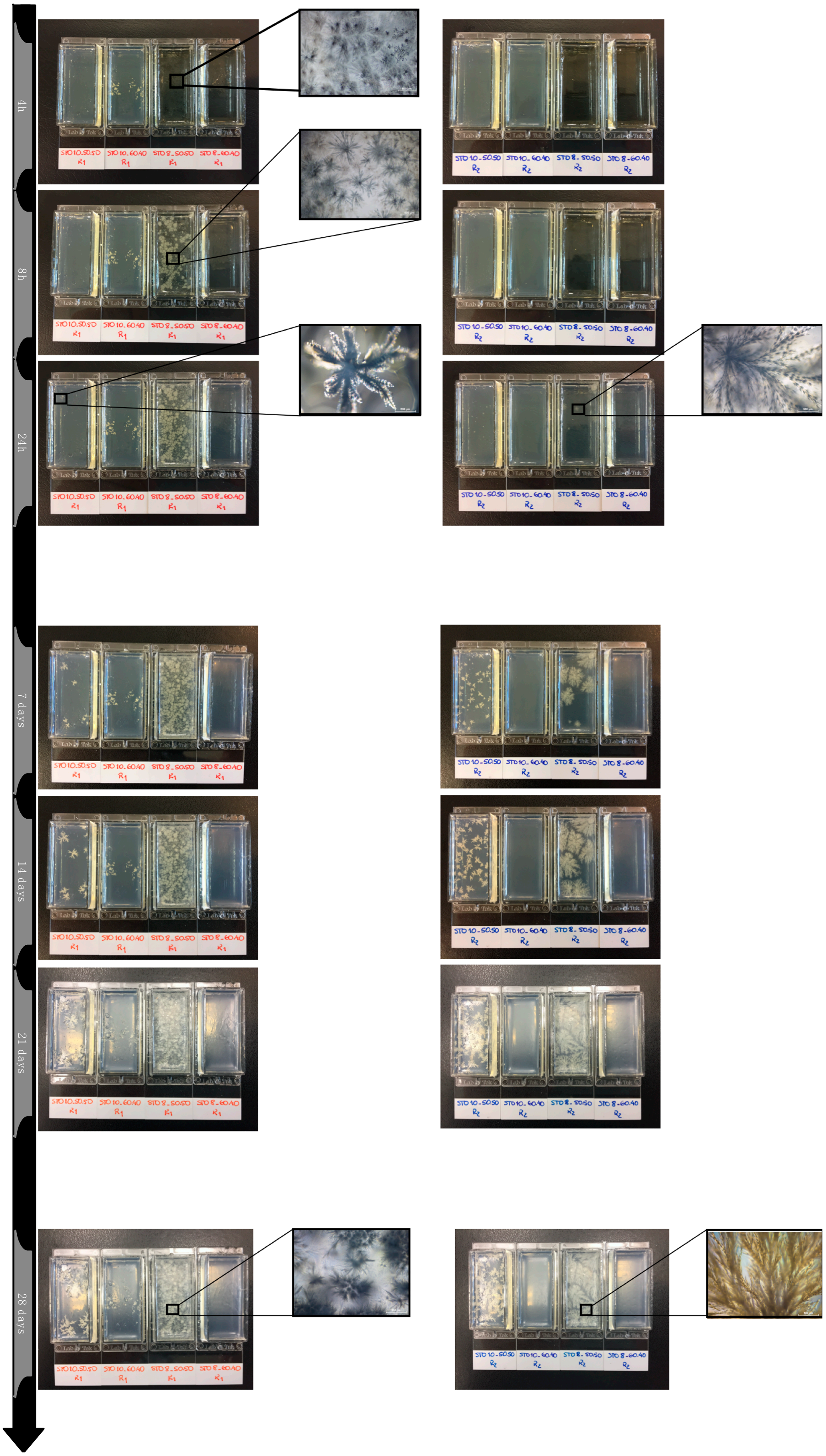
4 h
8 h
24 h
7 days
14 days
21 days
28 days

Figure S2. Pictures and polarized micrographs of oleogel samples registered at different time-points during the period of storage. Micrographs numbered from 1 to 6 refer to the polarized images obtained under the microscope with 100× magnification.

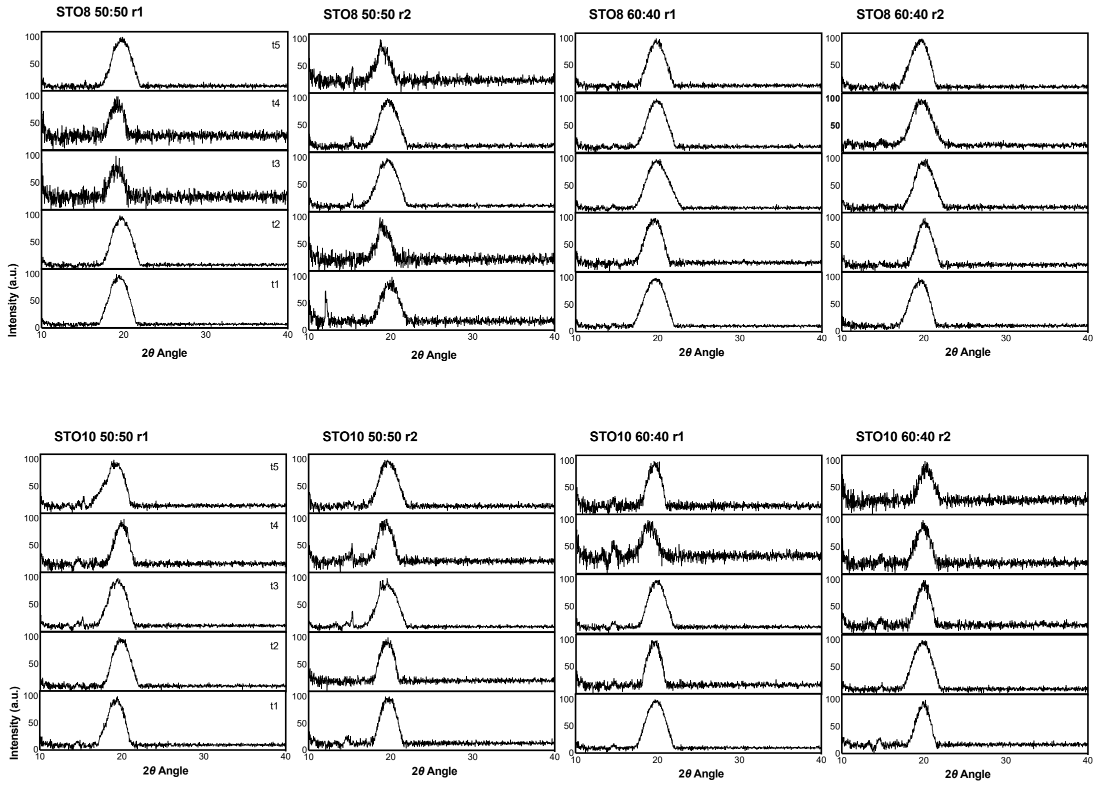

Figure S3. XRD spectra of STO samples during the storage period. Different points are displayed in growing order (bottom to top) from t1 to t5, where t1 = 24 h; t2 = 7 days; t3 = 2 weeks; t4 = 3 weeks; t5 = 28 days (4 weeks);.

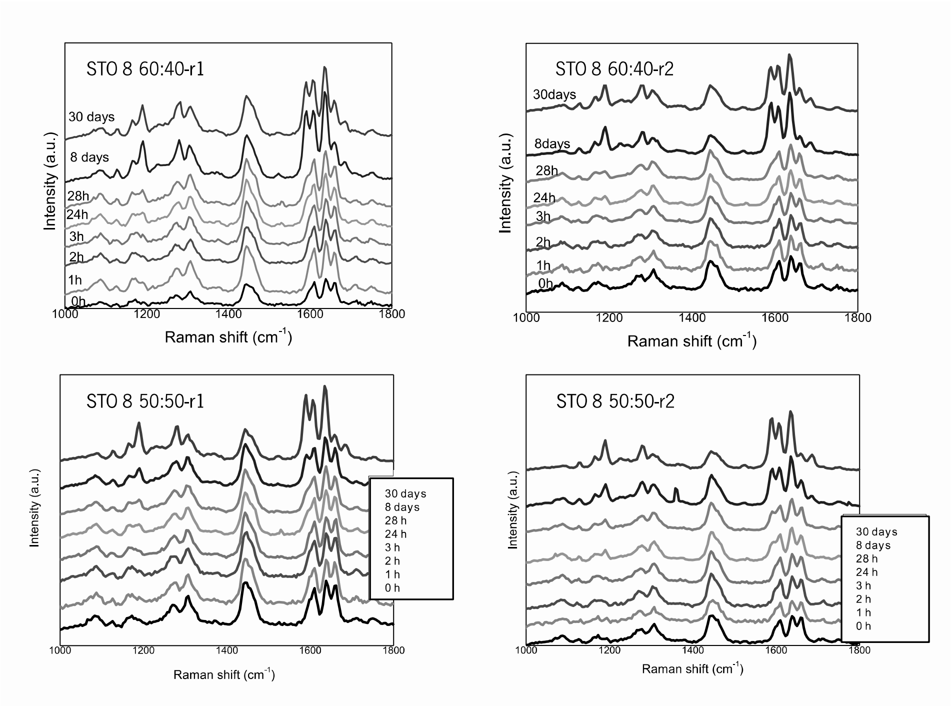

Figure S4. Raman spectra for STO8 samples produced with both r1 and r2 cooling ramps. The row above shows STO8 60:40 samples and below are portrayed the STO8 50:50 samples.

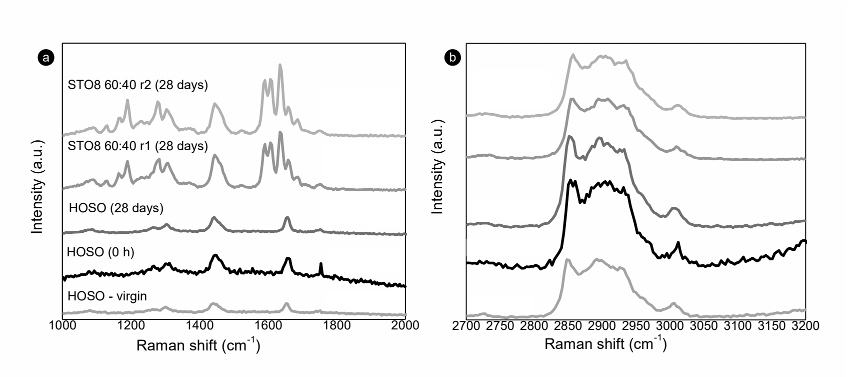

Figure S5. Raman spectra for oleogel and oil samples. (**a**) Raman spectra from 1000 to 2000 cm^{-1} for oil samples (HOSO virgin unheated; heated HOSO t28; STO8 60:40-r1 t28 days and STO8 60:40-r2 t28 days); (**b**), Raman spectra from 2700 to 3100 cm^{-1} for oil samples (HOSO virgin unheated; heated HOSO t0, heated HOSO t28 days, STO8 60:40-r1 t28 days nd STO8 60:40-r2 t28 days);.

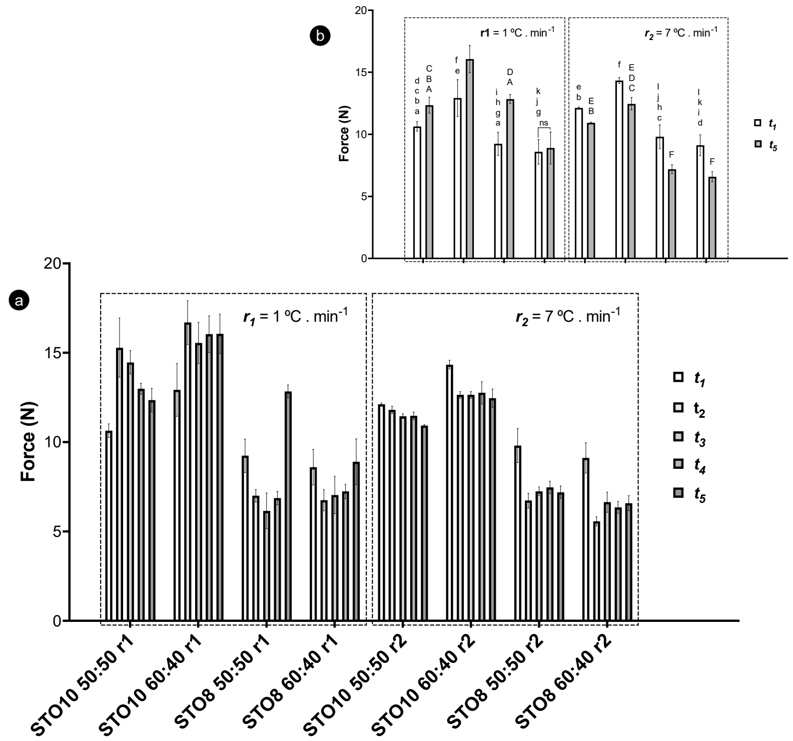

Figure S6. Hardness values of oleogels: (**a**) All oleogel formulations where t1 = 24 h; t2 = 7 days; t3 = 2 weeks; t4 = 3 weeks; t5 = 28 days (4 weeks). (**b**) Zoom in on the hardness values for t1 = 24 h and t5= 28 days (4 weeks). Tukey's multiple comparisons test results are displayed, same letters mean no difference among samples, small letters showing significance in differences between samples at t1 and capital letters showing significance in differences between samples at t5; ns shows the only non-significant result between time for the same sample.
